# Quercetin Enhances the Anti-Tumor Effects of BET Inhibitors by Suppressing hnRNPA1

**DOI:** 10.3390/ijms20174293

**Published:** 2019-09-02

**Authors:** Thao N.D. Pham, Sophie Stempel, Mario A. Shields, Christina Spaulding, Krishan Kumar, David J. Bentrem, Maria Matsangou, Hidayatullah G. Munshi

**Affiliations:** 1 Department of Medicine, Feinberg School of Medicine, Northwestern University, Chicago, IL 60611, USA; 2 Jesse Brown VA Medical Center, Chicago, IL 60612, USA; 3 The Robert H. Lurie Comprehensive Cancer Center, Chicago, IL 60611, USA; 4 Department of Surgery, Feinberg School of Medicine, Northwestern University, Chicago, IL 60611, USA

**Keywords:** flavonoids, quercetin, bromodomain and extraterminal domain (BET) inhibitors, combination therapy, pancreatic cancer, thyroid cancer, heterogeneous nuclear nucleoprotein A1 (hnRNPA1), survivin

## Abstract

Bromodomain and extraterminal domain (BET) proteins, which are important epigenetic readers, are often dysregulated in cancer. While a number of BET inhibitors are currently in early phase clinical trials, BET inhibitors show limited single-agent activity. The purpose of this study is to determine if Quercetin, a naturally occurring polyphenolic flavonoid often found abundant in fruits and vegetables, can enhance the anti-tumor effects of BET inhibitors. The efficacy of the combination was evaluated in vitro and in a xenograft model of pancreatic cancer. Co-treatment with BET inhibitors and Quercetin promoted apoptosis, decreased sphere-forming ability by cancer cells, and decreased cell proliferation. We found that hnRNPA1, a nuclear protein known to control mRNA export and mRNA translation of anti-apoptotic proteins, mediates some anti-tumor effects by Quercetin. Additionally, we show that combining BET inhibitors with Quercetin or hnRNPA1 knockdown decreased the anti-apoptotic protein Survivin. Significantly, Quercetin decreased hnRNPA1 in vivo and enhanced the effects of BET inhibitors at suppressing tumor growth. Together, these results demonstrate that Quercetin enhances the efficacy of BET inhibitors by suppressing hnRNPA1, and identify combination therapy with Quercetin and BET inhibitors for the treatment of cancer patients.

## 1. Introduction

The use of natural phytochemicals in anticancer regimens has gained much interest due to their widespread availability and their established safety profile. These natural compounds can promote apoptosis and autophagy in cancer cells and suppress the cancer stem cell population [1]. They can also enhance the efficacy of different cancer drugs. For example, Curcumin and Resveratrol have been reported to sensitize cancer cells to conventional chemotherapy drugs such as cisplatin [2,3,4], paclitaxel [5,6], and doxorubicin [7,8,9,10]. There is also increasing interest in evaluating the ability of these natural compounds to enhance the efficacy of novel cancer therapeutics.

Bromodomain and extraterminal domain (BET) proteins are important epigenetic readers that are involved in cancer pathogenesis. In recent years, BET inhibitors have been developed as anticancer agents with promising preclinical data. However, BET inhibitors show limited single-agent activity in early-phase clinical trials [11,12]. Also, resistance to BET inhibitors has been reported in pre-clinical models [12]. For example, cancer cells that develop resistance to BET inhibitors activate alternative signaling pathways to up-regulate oncogene expression [13,14,15]. Thus, there is increasing interest in identifying combination regimens to enhance the efficacy of BET inhibitors. We have previously reported combination therapy with BET and MNK inhibitors results in synergistic inhibition of thyroid and pancreatic cancer cells [16]. It is not known if natural flavonoids, such as Quercetin, can potentiate the efficacy of BET inhibitors.

Quercetin is a naturally occurring polyphenolic flavonoid which is found in many grains, fruits and vegetables. There is increasing evidence demonstrating that Quercetin has efficacy against multiple cancers, including lung [17], breast [18,19,20], prostate [21,22], and pancreatic [23,24] cancers. Quercetin modulates a number of signaling pathways to cause cell cycle arrest [17,18,19] and induce apoptosis [17,18,20]. Quercetin also synergizes with several agents [25,26,27,28,29] for enhanced anti-tumor effects. For example, Quercetin sensitizes resistant prostate tumors to the antiandrogen enzalutamide by targeting hnRNPA1 (heterogeneous nuclear nucleoprotein A1) [29], a nuclear protein involved in the regulation of alternative splicing, mRNA export, and mRNA translation [30,31]. hnRNPA1, which is overexpressed in a number of cancers, contributes to tumor progression [32,33]. hnRNPA1 mediates its anti-tumor effects in part by controlling mRNA export and mRNA translation of anti-apoptotic proteins XIAP and BCL-XL [34,35].

In this report, we provide data showing that Quercetin enhances the potency of BET inhibitors. We identify hnRNPA1 as a target of Quercetin and show that hnRNPA1 knockdown mirrors the effects of Quercetin when combined with BET inhibitors. Significantly, we demonstrate that Quercetin decreases hnRNPA1 and enhances the anti-tumor effects of BET inhibitors in vivo. Importantly, we show co-expression of hnRNPA1 with the BET protein BRD4 (Bromodomain-containing protein 4) in human tumor samples. Together, these results demonstrate that Quercetin enhances the efficacy of BET inhibitors by suppressing hnRNPA1, and identify combination therapy with Quercetin and BET inhibitors for the treatment of cancer patients.

## 2. Results

### 2.1. Quercetin Enhances the Anti-Tumor Effects of BET Inhibitors

**Figure 1:**
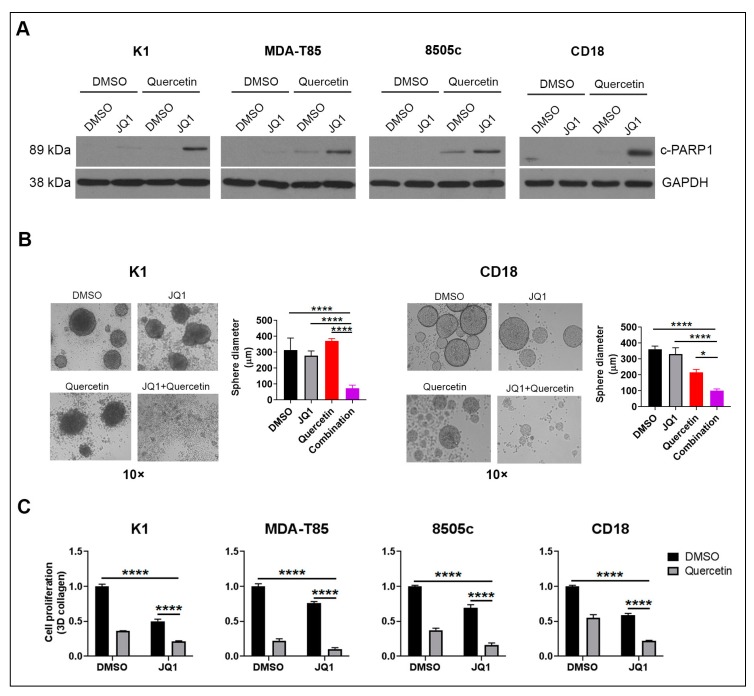
Quercetin enhances the anti-tumor effects of bromodomain and extraterminal domain (BET) inhibitors. (**A**) Thyroid and pancreatic cancer cells were treated with DMSO (control), JQ1 (1 µmol/L), Quercetin (20 µmol/L), or a combination of JQ1 (1 µmol/L) and Quercetin (20 µmol/L) for 24 h. The effect on apoptosis was determined by analyzing for c-PARP1 by western blot analysis, using GAPDH as loading control. (**B**) Thyroid K1 and pancreatic CD18 cancer cells were grown in suspension cultures in the presence of DMSO, JQ1 (1 µmol/L), Quercetin (20 µmol/L), or a combination of JQ1 (1 µmol/L) and Quercetin (20 µmol/L) for 72 h. The ability of cancer cells to form tumor spheres was examined by phase microscopy. The results are representative of at least three independent experiments. Error bars represent SD from at least three technical replicates. Two-way ANOVA analysis was performed. Comparison was made against the combined treatment group. *, *p* < 0.05; ****, *p* < 0.0001. (**C**) Thyroid and pancreatic cancer cells growing in 3D collagen were treated with DMSO, JQ1 (1 µmol/L), Quercetin (20 µmol/L), or combination of JQ1 (1 µmol/L) and Quercetin (20 µmol/L) for 72 h. The effect on proliferation was determined by WST-1 assay. Two-way ANOVA analysis was performed. ****, *p* < 0.0001. Error bars represent SD from three technical replicates. Results are representative of two to four independent experiments. DMSO: dimethyl sulfoxide; JQ1: BET inhibitor; c-PARP1: cleaved PARP; GAPDH: Glyceraldehyde 3-phosphate dehydrogenase (loading control); K1, MDA-T85: human papillary thyroid cancer cell lines; 8505c: human anaplastic thyroid cancer cell line; CD18: human pancreatic cancer cell line; SD: standard deviation; WST-1: tetrazolium salt converted by cellular mitochondrial dehydrogenases to formazan.

We first evaluated the effects of co-treatment with Quercetin and BET inhibitors in cancer cells. In thyroid and pancreatic cancer cells, co-treatment with Quercetin and BET inhibitor JQ1 significantly enhanced apoptosis (Figure 1A) and decreased sphere-forming ability (Figure 1B). In agreement with the existing reports on the anti-proliferative effects of Quercetin [17,18,19], we found that Quercetin inhibited proliferation of thyroid and pancreatic cancer cells (Figure 1C). Importantly, the combination of Quercetin and JQ1 was more effective than single-agent treatment at suppressing proliferation of cancer cells (Figure 1C and Table S1). In addition, we observed similar results using the BET inhibitor OTX-015 (Birabresib) (Figure S1A,B and Table S1), which is currently in clinical trials for hematologic malignancies and select solid tumors [12].

### 2.2. hnRNPA1 Knockdown Enhances the Anti-Tumor Effects of BET Inhibitors

**Figure 2:**
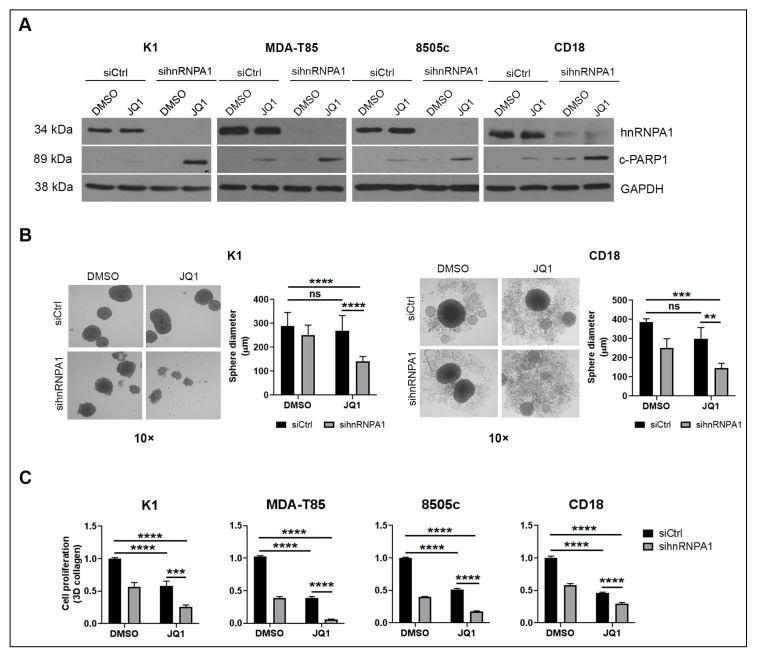
hnRNPA1 knockdown enhances the anti-tumor effects of BET inhibitors in cancer cells. (**A**) Cancer cells were transfected with siCtrl or sihnRNPA1 for 48 h, and then treated with JQ1 (1 µmol/L) for additional 24 h. Expression of hnRNPA1, c-PARP1 and GAPDH (loading control) were evaluated by western blot analysis. (**B**) Thyroid K1 and pancreatic CD18 cancer cells were transfected with siCtrl or sihnRNPA1 for 48 h, collected and grown in suspension cultures in the presence of DMSO or JQ1 (1 µmol/L) for additional 72 h. The ability of cancer cells to form tumor spheres was examined by phase microscopy. The results are representative of at least three independent experiments. Error bars represent SD from at least three technical replicates. Two-way ANOVA analysis was performed. **, *p* < 0.01; ***, *p* < 0.001; ****, *p* < 0.0001; ns, non-significant. (**C**) Thyroid and pancreatic cancer cells were transfected with control siRNA (siCtrl) or hnRNPA1-targeting siRNA (sihnRNPA1) for 48 h. The cells were then embedded in 3D collagen, treated with JQ1 (1 µmol/L) for additional 48 h, and the effect on cell proliferation was determined using the WST-1 assay. Two-way ANOVA analysis was performed. ***, *p* < 0.001 ****, *p* < 0.0001. Error bars represent SD from three technical replicates. Results are representative of at least three independent experiments. hnRNPA1: heterogeneous nuclear ribonucleoprotein A1; DMSO: dimethyl sulfoxide; JQ1: BET inhibitor; c-PARP1: cleaved PARP1; GAPDH: Glyceraldehyde 3-phosphate dehydrogenase (loading control); K1, MDA-T85: human papillary thyroid cancer cell lines; 8505c: human anaplastic thyroid cancer cell line; CD18: human pancreatic cancer cell line; SD: standard deviation; WST-1: tetrazolium salt converted by cellular mitochondrial dehydrogenases to formazan.

We recently reported that targeting MNK kinases enhances the anti-proliferative effects of BET inhibitors in cancer cells [16]. Since hnRNPA1 is a MNK effector [36], and is inhibited by Quercetin in prostate cancer cells [29], we evaluated whether hnRNPA1 knockdown also enhances the anti-tumor effects of BET inhibitors. Thyroid and pancreatic cancer cells were transfected with hnRNPA1 siRNA and co-treated with BET inhibitors. Knockdown of hnRNPA1 and concurrent treatment with BET inhibitor JQ1 led to increased apoptosis (Figure 2A). Moreover, hnRNPA1 knockdown enhanced the effects of JQ1 at suppressing sphere-forming ability of cancer cells (Figure 2B). Similar to Quercetin treatment, knockdown of hnRNPA1 decreased proliferation and enhanced the anti-proliferative effects of JQ1 compared to control siRNA (Figure 2C and Table S1). Importantly, hnRNPA1 knockdown also enhanced the effects of the BET inhibitor OTX-015 at inducing apoptosis and suppressing proliferation of cancer cells (Figure S1C,D and Table S1).

### 2.3. hnRNPA1 Mediates the Anti-Tumor Effects of Quercetin

**Figure 3:**
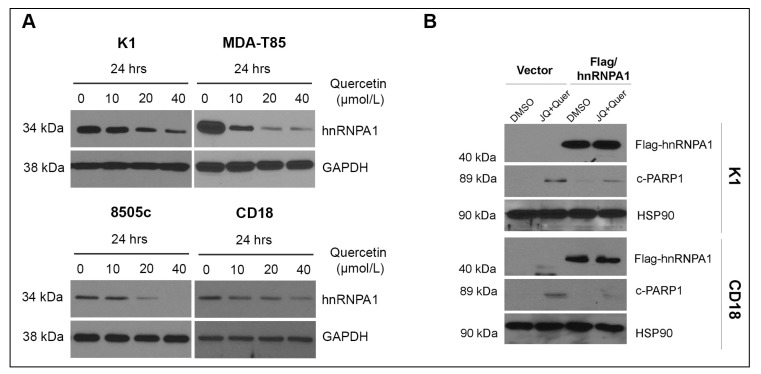
hnRNPA1 mediates the anti-tumor effects of Quercetin. (**A**) Thyroid and pancreatic cancer cells were treated with increasing concentrations of Quercetin for 24 h, and the cells were analyzed for hnRNPA1 and GAPDH (loading control) by western blot analysis. (**B**) Cancer cells transfected with control vector or vector expressing hnRNPA1 were treated with DMSO or a combination of JQ1 (JQ, 1 µmol/L) and Quercetin (Quer, 20 µmol/L) for 24 h. Expression of flag-tagged hnRNPA1 and the effect on c-PARP-1 was determined by western blot analysis using HSP90 as loading control. The results are representative of three independent experiments. hnRNPA1: heterogeneous nuclear ribonucleoprotein A1; GAPDH: Glyceraldehyde 3-phosphate dehydrogenase (loading control); c-PARP1: cleaved PARP1, HSP90: heat shock protein 90 (loading control); DMSO: dimethyl sulfoxide; JQ1: BET inhibitor; K1, MDA-T85: human papillary thyroid cancer cell lines; 8505c: human anaplastic thyroid cancer cell line; CD18: human pancreatic cancer cell line.

Since Quercetin suppresses hnRNPA1 levels in prostate cancer cells [29], we evaluated whether hnRNPA1 mediates the anti-tumor effects of Quercetin in thyroid and pancreatic cancer cells. Quercetin decreased hnRNPA1 protein levels in a dose- (Figure 3A) and a time-dependent (Figure S2A) manner in both thyroid and pancreatic cancer cells. Importantly, Quercetin did not affect hnRNPA1 mRNA levels in thyroid and pancreatic cancer cells (Figure S2B). To demonstrate that Quercetin enhanced the pro-apoptotic effects of BET inhibitors by decreasing hnRNPA1, we overexpressed hnRNPA1 and evaluated the effects of co-treatment with Quercetin and BET inhibitors on apoptosis. hnRNPA1 overexpression decreased PARP1 cleavage induced by co-treatment with JQ1 and Quercetin, indicating that the apoptotic effects of Quercetin are mediated in part by Quercetin suppression of hnRNPA1 (Figure 3B).

### 2.4. Co-Treatment with Quercetin and JQ1 Decreases Survivin

**Figure 4:**
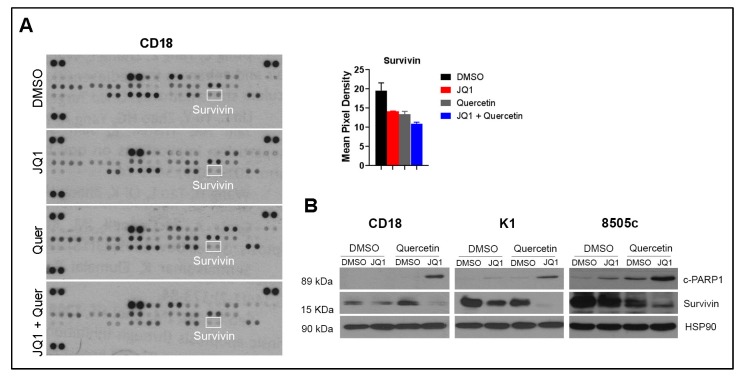
Co-treatment of Quercetin and JQ1 decreases Survivin. (**A**) Pancreatic cancer cells were treated with DMSO, JQ1 (1 µmol/L), Quercetin (Quer, 20 µmol/L), or a combination of JQ1 (1 µmol/L) and Quercetin (20 µmol/L) for 24 h. Cell lysates were collected and analyzed for apoptosis-related proteins using the Proteome Profiler Human Apoptosis Array ARY009, and the pixel density of Survivin from the array data was quantified by ImageJ. Error bars represent SD from two technical replicates. The results are representative of two independent experiments. Survivin is highlighted with white boxes. (**B**) Cells were treated with DMSO, JQ1 (1 µmol/L), Quercetin (20 µmol/L), or a combination of JQ1 (1 µmol/L) and Quercetin (20 µmol/L) for 24 h. The effect on c-PARP1 and Survivin was determined by western blot analysis, using HSP90 as loading control. The results are representative of three independent experiments. DMSO: dimethyl sulfoxide; JQ1: BET inhibitor; c-PARP1: cleaved PARP1; HSP90: heat shock protein 90 (loading control); SD: standard deviation; K1: human papillary thyroid cancer cell lines; 8505c: human anaplastic thyroid cancer cell line; CD18: human pancreatic cancer cell line.

We next evaluated the effects of treatment with Quercetin and JQ1 on pro- and anti-apoptotic proteins using the proteome profiler apoptosis array ARY009. There were minimal effects with single-agent treatment with JQ1 or Quercetin, or with the combination of JQ1 and Quercetin, on the expression of several apoptosis-regulating proteins such as Bad, Bax, and Bcl-2 in CD18 and K1 cells (Figure S3). While single-agent treatment with Quercetin and JQ1 had minimal effects on Survivin protein levels in the array, co-treatment with Quercetin and JQ1 suppressed Survivin in these cell lines (Figure 4A and Figure S3). We also evaluated the effects of Quercetin and JQ1 on Survivin protein levels by western blot analysis (Figure 4B). While Quercetin decreased Survivin levels in thyroid cancer cells, but not in CD18 pancreatic cancer cells, co-treatment with JQ1 and Quercetin resulted in pronounced reduction in Survivin levels in both thyroid and pancreatic cancer cells (Figure 4B).

### 2.5. Co-Treatment with hnRNPA1 Knockdown and JQ1 Decreases Survivin

**Figure 5:**
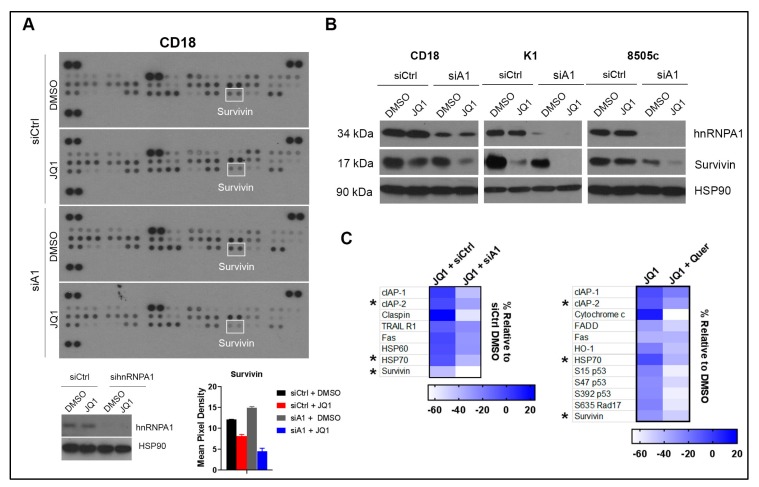
Combination of hnRNPA1 knockdown and JQ1 decreases Survivin. (**A**) CD18 pancreatic cancer cells were transfected with control siRNA (siCtrl) or hnRNPA1-targeting siRNA (sihnRNPA1, siA1) for 48 h. The cells were then treated with DMSO or JQ1 (1 µmol/L) for an additional 24 h. Cell lysates were collected and analyzed for apoptosis-related proteins using Proteome Profiler Human Apoptosis Array ARY009, and the pixel density of Survivin from the array data, highlighted with white boxes, was quantified by ImageJ. hnRNPA1 knockdown was confirmed by western blot analysis. Error bars represent SD from two technical replicates. The results are representative of two independent experiments. (**B**) Cancer cells transfected with siCtrl or sihnRNPA1 (siA1) for 48 h were subsequently treated with DMSO or JQ1 (1 µmol/L) for an additional 24 h. Cell lysates were analyzed for expression of hnRNPA1 and Survivin by western blot analysis, using HSP90 as loading control. (**C**) Average pixel density from two independent experiments with CD18 cells was calculated for each protein in the array and normalized to the corresponding control treatment group. Heat maps reflect % change in the expression of select proteins in cells transfected with siCtrl or sihnRNPA1 (siA1) and co-treated with JQ1 and in cells treated with JQ1 alone or a combination of JQ1 and Quercetin (Quer). *, proteins common in both heat maps. DMSO: dimethyl sulfoxide; JQ1: BET inhibitor; HSP90: heat shock protein 90 (loading control); hnRNPA1: heterogeneous nuclear ribonucleoprotein A1; cIAP-2: cellular inhibitor of apoptosis protein 2; HSP70: heat shock protein 70; SD: standard deviation; K1: human papillary thyroid cancer cell line; 8505c: human anaplastic thyroid cancer cell line; CD18: human pancreatic cancer cell line.

We also evaluated the effects of hnRNPA1 knockdown and JQ1 treatment on the pro- and anti-apoptotic proteins using the apoptosis array (catalog #ARY009). hnRNPA1 knockdown and JQ1 treatment suppressed Survivin in cancer cells (Figure 5A), which was validated by western blot analysis (Figure 5B). Combination of hnRNPA1 knockdown and JQ1 also decreased Survivin levels in thyroid cancer cells (Figure 5B). Importantly, we found that hnRNPA1 knockdown enhances the ability of JQ1 at suppressing expression of several anti-apoptotic proteins (Figure 5C and Table S2). Significantly, many of the same proteins were also found to be suppressed by co-treatment with Quercetin and JQ1 (Figure 5C and Table S3). Compared to JQ1 treatment alone, JQ1 in combination with either hnRNPA1 knockdown or Quercetin treatment further downregulated protein levels of cIAP-2 (cellular inhibitor of apoptosis protein 2), HSP70 (heat shock protein 70) and Survivin.

### 2.6. Quercetin Enhances the Anti-Tumor Effects of JQ1 In Vivo

**Figure 6:**
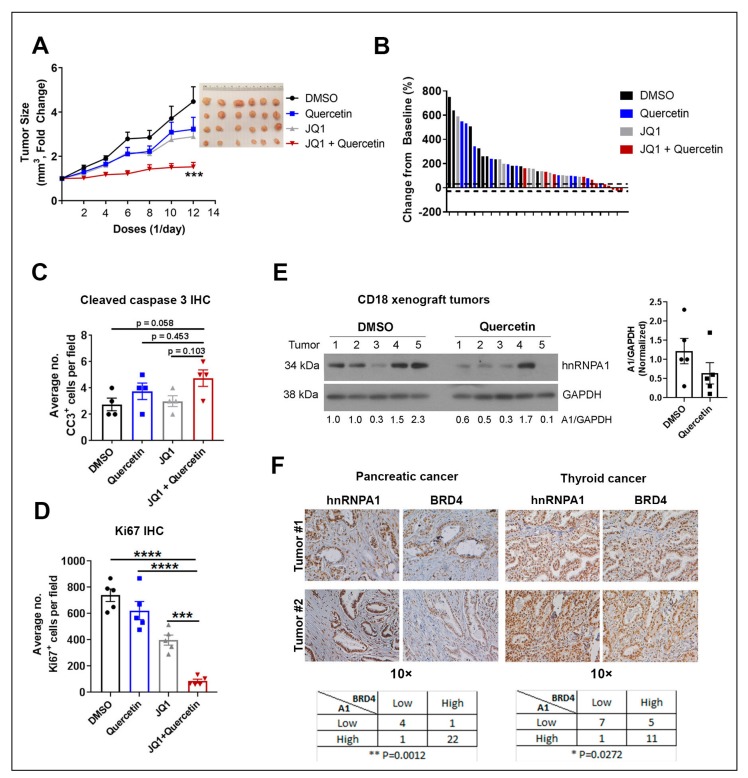
Quercetin increases the anti-tumor effects of JQ1 in vivo. CD18 pancreatic cancer cells (1.5 × 10^6^) were subcutaneously injected into the flanks of nude mice and allowed to form tumors for 2 weeks. Mice with established tumors were randomized and treated intraperitoneally with DMSO, JQ1 (50 mg/kg), Quercetin (40 mg/kg), or a combination of JQ1 (50 mg/kg) and Quercetin (40 mg/kg) daily Mon–Fri for 3 weeks. (**A**) Tumor growth was assessed daily Mon–Fri, and tumor volume calculated and normalized to tumor volume at the start of treatment. ***, *p* < 0.001 compared to the DMSO-treated group. Error bars represent SEM from at least 10 individual tumors. (**B**) Waterfall plot was generated to demonstrate change in tumor volume relative to start of treatment. Dotted lines indicate 30% cut-off values from the baseline. (**C**) The tumor specimens were stained for the apoptosis marker cleaved caspase-3 (CC3) by immunohistochemistry and the number of CC3^+^ cells per 10× field were counted. At least five different sections per individual tumor were counted. Four tumors from each treatment group were used in the analysis. Error bars represent SEM from all tumors analyzed. (**D**) The tumor specimens were stained for proliferation marker Ki67 by immunohistochemistry and the number of Ki67^+^ cells per 10× field were counted by ImageJ analysis. At least four different sections per individual tumor were counted. Five tumors from each treatment group were used in the analysis. Two-way ANOVA analysis was performed. ***, *p* < 0.001 ****, *p* < 0.0001. Error bars represent SEM from all tumors analyzed. (**E**) The expression of hnRNPA1 (A1) in CD18 tumors was analyzed by western blot analysis, with GAPDH as loading control. Densitometry graph is shown. Error bars represent SEM from five individual tumors. (**F**) TMA generated from pancreatic ductal adenocarcinoma (PDAC) (*n* = 28) and human papillary thyroid cancer (*n* = 24) specimens were stained for BRD4 and hnRNPA1 by immunohistochemistry, and the relative staining was graded as low (0 or 1+) or high (2+ or 3+). Fisher’s exact test was used to evaluate the relationship between BRD4 and hnRNPA1 (A1) in these specimens. DMSO: dimethyl sulfoxide; JQ1: BET inhibitor; hnRNPA1: heterogeneous nuclear ribonucleoprotein A1; GAPDH: Glyceraldehyde 3-phosphate dehydrogenase (loading control); BRD4: bromodomain-containing protein 4; IHC: immunohistochemistry; TMA: tissue microarray; CD18: human pancreatic cancer cell line.

To evaluate whether Quercetin can enhance the anti-tumor effects of JQ1 in vivo, pancreatic cancer cells were injected into nude mice and allowed to form tumors for two weeks. Established tumors were then treated with vehicle control (DMSO), JQ1, Quercetin, or a combination of JQ1 and Quercetin. Co-treatment with Quercetin and JQ1 was more effective in suppressing tumor growth compared to single-agent treatment (Figure 6A). Waterfall plot analysis of the individual tumors also showed that the combination treatment was more effective than treatment with either Quercetin or JQ1 at limiting tumor growth (Figure 6B). Importantly, the combination treatment was well-tolerated (Figure S4). To assess the effects on tumor apoptosis and proliferation, we stained the tumors for cleaved caspase-3 (CC3) and Ki67. While there were minimal effects on CC3 staining in the tumor specimens (Figure 6C and Figure S5), combination of Quercetin and JQ1 was significantly more effective than single-treatment at suppressing tumor proliferation (Figure 6D and Figure S5). Importantly, consistent with the in vitro findings, we found that Quercetin decreased hnRNPA1 in vivo (Figure 6E).

### 2.7. Expression of hnRNPA1 and BRD4 in Human Tumor Specimens

Since the effects of BET inhibitors are often through inhibition of the BET protein BRD4 [11,12,37,38], we evaluated expression of hnRNPA1 and BRD4 in human tumor specimens. We found significant co-expression of these two proteins in human pancreatic (*p* = 0.0012) and thyroid (*p* = 0.0272) specimens (Figure 6F), providing additional rationale for co-targeting hnRNPA1 and BRD4 using the combination of Quercetin and BET inhibitors.

## 3. Materials and Methods

### 3.1. Cell Culture

Human thyroid cancer cell lines K1 and 8505c were purchased from Sigma-Aldrich (St. Louis, MO, USA) and cultured in Dulbecco’s Modified Eagle Medium (DMEM):Ham′s F12:MCDB 105 in a 2:1:1 ratio and Roswell Park Memorial Institute (RPMI)-1640 media, respectively. Human thyroid cancer cell line MDA-T85 was obtained from MD Anderson Cancer Center and was cultured in RPMI-1640 as previously described [39]. Human pancreatic ductal adenocarcinoma cell line CD18 was obtained from the American Type Culture Collection (ATCC, Manassas, VA, USA) and was maintained in DMEM [40]. All media contained 10% Fetal Bovine Serum (FBS) and antibiotics (100 U/mL Penicillin and 100 µg/mL Streptomycin). All human cell lines have been authenticated using Short Tandem Repeat profiling within the last three years. All cell lines used are listed using the official cell line name and its Research Resource Identifier (RRID) as available in the ExPASy Cellosaurus database. Cell lines were tested mycoplasma free in 2019.

### 3.2. Chemicals

JQ1 [37] was purchased from Tocris Bioscience (Minneapolis, MN, USA); OTX-015 [38] was obtained from Selleckchem (Houston, TX, USA); and Quercetin [29] (≥95%, HPLC) was obtained from Sigma-Aldrich. Drugs were dissolved in solvent according to the manufacturer’s instruction and stored at −20 °C.

### 3.3. Embedding Cells in Three-Dimensional Type I Collagen Gels

Cancer cells were suspended in type I collagen solution as described previously [41]. Briefly, collagen type I rat tail protein (Corning) was diluted to 2.2 mg/mL with DMEM, water, and NaOH and allowed to gel for 15 min at 37 °C. For morphologic examination of cells, cell colonies in three-dimensional collagen were examined using a Zeiss Axiovert 40 CFL microscope and pictures taken with a Nikon Coolpix 4500 camera.

### 3.4. Sphere Forming Assay

Cancer cells were trypsinized and seeded in agar-coated plates to prevent attachment. Cells were cultured in DMEM/F12 (Invitrogen, Waltham, MA, USA), supplemented with B27 (2%), Epidermal Growth Factor (EGF) (20 ng/mL, in H_2_O, 1% Bovine Serum Albumin (BSA), Peprotech, Rocky Hill, NJ, USA), basic Fibroblast Growth Factor (FGF) (20ng/mL, Peprotech, in 5 mM Tris pH 7.6, 0.1% BSA), and drug treatment of choice. After 3 days, sphere number and size were examined under a Zeiss Axiovert 40 CFL microscope and pictures were taken with a Nikon Coolpix 4500 camera for further analysis.

### 3.5. WST-1 Proliferation Assay

As described previously [41], approximately 1000 cells were embedded in 2.2 mg/mL collagen I solution in 96-well plates. After 3 days of treatment with the different inhibitors, WST-1 reagent (Sigma-Aldrich) was added into the media at a 1:100 dilution factor. Absorbance was measured according to the manufacturer’s instructions every hour for 4 h or until maximal absorbance was reached.

### 3.6. Transfection

siRNA targeting hnRNPA1 was obtained from Ambion. All siRNA transfections were carried out using RNAimax reagent (Life Technology, Rockville, MD, USA) according to the manufacturer’s instructions. hnRNPA1 clone (transcript variant 1, RC203314) was obtained from Origene (Rockville, MD, USA), verified by sequencing, and transfected into cells with Lipofectamine 3000 (Life Technology) according to the manufacturer’s instructions.

### 3.7. Immunoblotting

Whole cell extracts of cultured cells were prepared in radioimmunoprecipitation (RIPA) lysis buffer supplemented with phosphatase and protease inhibitors (Calbiochem, Burlington, MA, USA). Protein concentration was determined by the bicinchoninic acid assay (BCA) (Thermo Fisher Scientific, Waltham, MA, USA) and separated by SDS-PAGE gel. The following antibodies and dilutions were used: hnRNPA1 (1:2000, Cell Signaling, Danvers, MA, USA), cleaved PARP1 (1:1000, Cell Signaling), HSP90 (1:4000, Santa Cruz Biotechnology, Santa Cruz, CA, USA), GAPDH (1:3000, EMD Millipore, Billerica, MA, USA), Survivin (1:2000, Cell Signaling), and Flag-tag (1:2000, Sigma-Aldrich). Following blocking with 5% BSA, membranes were incubated with the primary antibodies overnight at 4 °C. The next day, membrane was incubated with secondary antibodies for 1 h at room temperature. Secondary anti-mouse IgG (A4416) and anti-rabbit IgG (A6667) antibodies were purchased from Sigma-Aldrich and used at a 1:4000 dilution. Images of blots were acquired on HyBlot ES Autoradiography Film (Thomas Scientific, Swedesboro, NJ, USA) following incubation with SuperSignal West Pico PLUS (Thermo Fisher Scientific). When necessary, membranes were stripped using Restore Western Blot Stripping Buffer (Thermo Fisher Scientific) according to manufacturer’s instructions.

### 3.8. Immunohistochemistry

Human thyroid tissue microarrays (TMAs) were purchased from US Biomax (Rockville, MD, USA), while human PDAC TMAs were created by the Pathology Core Facility at Northwestern University from de-identified PDAC specimens. The TMAs were stained for BRD4 (Rabbit, Abcam, Cambridge, United Kingdom) and hnRNPA1 (Rabbit, Abcam). CD18 tumors were stained for Ki67 (Rabbit, Cell Signaling) and cleaved caspase 3 (Rabbit, Cell Signaling). Antigen retrieval was carried out as previously described [42,43]. Photographs for quantitative comparison were taken using FeinOptic microscope and Jenoptik ProgRes C5 camera.

### 3.9. Proteome Profiler Human Apoptosis

The membrane-based immunoassay was purchased from R&D Systems (Minneapolis, MN, USA) (catalog #ARY009). Whole cell extracts were collected, incubated with the membranes overnight, and analyzed for the expression of different apoptotic proteins following the manufacturer’s instructions. Each assay was repeated twice. Pixel density was quantified by ImageJ.

### 3.10. In Vivo Study

CD18 cancer cells were injected subcutaneously (1.5 × 10^6^ cells/site) into the flanks of 6–8-week-old female nu/nu mice (Taconic Biosciences, Rensselaer, New York, USA). When tumors achieved an approximate volume of 100 mm^3^, mice were randomized and treated with the following chemicals: control (Dimethyl Sulfoxide, DMSO), JQ1 (50 mg/kg), Quercetin (40 mg/kg), or a combination of JQ1 (50 mg/kg) and Quercetin (40 mg/kg). Treatments were administered daily Mon–Fri for 3 weeks in a suspension containing 10% hydroxypropyl-β-cyclodextrin in double-distilled water. Tumor volume was obtained using the formula *V* = (*W*^2^ × *L*)/2 where *V* is tumor volume, *W* is tumor width, *L* is tumor length by caliper measurement. The mice were euthanized by CO_2_ inhalation and cervical dislocation, and the tumors were excised and photographed. The number of cleaved caspase-3^+^ cells from at least five different 10× sections was manually counted. The number of Ki67^+^ cells from at least four different 10× sections was analyzed by ImageJ.

### 3.11. Statistical Analysis

Error bars represent standard deviation (SD) or standard error of the mean (SEM) as specified. All statistical analyses were done using GraphPad Instat. In vivo analysis for cleaved caspase-3 and Ki67 expression was performed using ordinary one-way ANOVA, followed by Dunnett’s multiple comparison test. A *p* value <0.05 was considered significant.

### 3.12. Study Approval

All animal work and procedures were approved by the Northwestern University Institutional Animal Care and Use Committee. In addition, all animal experiments were performed in accordance with relevant guidelines and regulations.

## 4. Discussion

Several independent reports have demonstrated promising pre-clinical activity of BET inhibitors [11,12]. Furthermore, results from early-phase clinical trials with OTX-015 (Birabresib) compound indicate that it is overall well tolerated, and the toxicities are reportedly reversible with treatment interruptions [12,44]. However, mechanisms of resistance to BET inhibitors have been reported [13,14,15], leading to increased interest in identifying combination regimens to enhance their efficacy. We recently published that in thyroid and pancreatic cancer, inhibitors targeting MNK kinases can enhance the efficacy of BET inhibitors by suppressing a pro-survival signaling pathway initiated by cancer cells following treatment with BET inhibitors [16]. In this report, we show that targeting the MNK effector hnRNPA1 also enhances the effects of BET inhibitors. Moreover, for the first time, we demonstrate that Quercetin, which suppresses hnRNPA1, can also enhance the anti-tumor effects of BET inhibitors in vitro and in vivo. The JQ1/Quercetin co-treatment significantly reduced tumor proliferation; however, the effect on apoptosis, as measured by cleaved caspase-3 staining, was minimal. These findings are in agreement with the tumor growth data, which demonstrate tumor stabilization rather than regression. This suggests that in the in vivo setting, the anti-tumor properties of the co-treatment rely more on its anti-proliferative, rather than the pro-apoptotic, effects.

Quercetin is a naturally occurring polyphenolic flavonoid that has been shown to have promising anti-cancer properties without noticeable toxicity in non-transformed or normal cells (reviewed in [45]). Quercetin has been shown to regulate a number of key molecular signaling pathways for its tumor suppressive effects. It can suppress NF-κB (Nuclear Factor Kappa Beta), PI3K (Phosphoinositide 3-kinases), Akt (also known as protein kinase B), and cyclin D1 [46,47,48,49], decrease expression of pro-survival proteins, and increase expression of pro-apoptotic proteins [50,51,52,53]. Importantly, Quercetin-induced apoptosis in glioma cells was through suppression of Survivin [54]. Here, we have found that Quercetin can decrease Survivin levels in thyroid cancer cells, but not in CD18 pancreatic cancer cells. However, co-treatment with BET inhibitors and Quercetin causes pronounced decrease in Survivin levels in both thyroid and pancreatic cancer cells. Importantly, targeting Survivin, which is upregulated in many tumors, can enhance apoptosis [55,56]. In addition, Quercetin can strongly suppress cancer stem cell proliferation and their ability to form tumorspheres [57]. Tumorsphere formation is commonly used to evaluate the self-renewal capability of cancer stem cells and to enrich this population from bulk tumor mass [58]. The effect of combination therapy with Quercetin and some known anti-cancer agents on tumorsphere formation has previously been studied. For example, Quercetin synergizes with epigallocatechin gallate, another popyphenolic compound, to suppress sphere-forming potential of CD44^+^/CD133^+^ prostate cancer cells [59]. In this study, we found that co-treatment with Quercetin and BET inhibitors significantly suppressed sphere-forming ability of pancreatic and thyroid cancer cells, suggesting that the combination may effectively target cancer stem cells.

Even though Quercetin can suppress tumor development through modulation of a number of signaling pathways, we show that Quercetin enhances the anti-tumor effects of JQ1 in part by suppressing hnRNPA1. hnRNPA1, the most ubiquitously expressed member of the hnRNP protein family, contributes to tumor development and progression [60]. While the role of hnRNPA1 in thyroid cancer progression has not been described, hnRNPA1 has previously been shown to regulate oncogenic Kras signaling in pancreatic cancer cells to mediate pancreatic cancer progression [61]. Here we show that hnRNPA1 knockdown, similar to Quercetin treatment, can enhance the effects of BET inhibitors on proliferation, apoptosis, and cell survival in both thyroid and pancreatic cancer cells. Overexpression of hnRNPA1 partially rescued the effects of combination treatment on apoptosis, demonstrating that hnRNPA1 mediates some of the effects of Quercetin. Furthermore, similar to Quercetin, hnRNPA1 knockdown enhances the ability of BET inhibitors at suppressing Survivin expression. We also identified additional proteins that were suppressed both when JQ1 was combined with Quercetin and when JQ1 was combined with hnRNPA1 knockdown. Taken together, these data suggest that the effects of Quercetin on JQ1-mediated apoptosis can be explained in part by Quercetin suppression of hnRNPA1.

Overall, we demonstrate that targeting of hnRNPA1 by Quercetin enhances the efficacy of BET inhibitors in thyroid and pancreatic tumors. Given co-expression of hnRNPA1 and the BET protein BRD4 in human thyroid and pancreatic tumors, and the favorable safety profiles of Quercetin and BET inhibitors, our study provides a rationale for pursuing this combination therapy in advanced cancer patients.

## Supplementary Materials

The following are available online at https://www.mdpi.com/1422-0067/20/17/4293/s1, Figure S1: Both Quercetin and hnRNPA1 knockdown enhance the anti-tumor effects of BET inhibitors, Figure S2: Quercetin decreases hnRNPA1 protein, Figure S3: Co-treatment of Quercetin and JQ1 decreases Survivin while having no effects on other apoptosis regulating proteins, Figure S4: The combination treatment of Quercetin and BET inhibitor JQ1 was well-tolerated, Figure S5: Representative IHC images for cleaved caspase-3 and Ki67 for CD18 tumors, Table S1: Calculations for coefficients of drug interaction, Table S2: Effect of hnRNPA1 knockdown on JQ1-mediated changes in proteins in the ARY009 apoptosis array, Table S3: Effect of Quercetin on JQ1-mediated changes in proteins in the ARY009 apoptosis array.
