# Supplementary material for: Quercetin Enhances the Anti-Tumor Effects of BET Inhibitors by Suppressing hnRNPA1
Source: Int J Mol Sci. 2019 Sep 2;20(17):4293. doi: 10.3390/ijms20174293 (PMC6747365; doi:10.3390/ijms20174293)
Supplement: Supplementary file 1 [file ijms-20-04293-s001.zip › ijms-558961-supplementary/ijms-558961-Sup Legend.docx]

**Quercetin enhances the anti-tumor effects of BET inhibitors by suppressing hnRNPA1**

**Thao N.D. Pham 1,4,*, Sophie Stempel 1, Mario Shields 1, Christina Spaulding 1,4, Krishan Kumar 1,3, David J. Bentrem 2,3,4, Maria Matsangou 1,3 and Hidayatullah G. Munshi 1,3,4,***

**Supplementary Data Legend**

**Figure S1**: Both Quercetin and hnRNPA1 knockdown enhance the anti-tumor effects of BET inhibitors. **A.** Cancer cells were treated with DMSO, OTX-015 (0.5 μmol/L), Quercetin (20 μmol/L), or a combination of OTX-015 (0.5 μmol/L) and Quercetin (20 μmol/L) for 24 hours. The cells were analyzed for cPARP-1 and GAPDH by western blot analysis. **B.**Cancer cells embedded in 3D collagen were treated with DMSO, OTX-015 (0.5 μmol/L), Quercetin (20 μmol/L), or a combination of OTX-015 (0.5 μmol/L) and Quercetin (20 μmol/L) for 72 hours. The effect on proliferation was determined by WST-1 assay. Two-way ANOVA analysis was performed. *, p<0.05 ****, p<0.0001. Error bars represent SD from three technical replicates. Results are representative of three independent experiments. **C.**Thyroid cancer cells K1 and 8505c were transfected with siCtrl or sihnRNPA1 for 48 hours and then treated with OTX-015 (0.5 μmol/L) for 24 hours. Expression of hnRNPA1, cPARP-1 and GAPDH were evaluated by western blot analysis. **D.** Thyroid cancer cells K1 and 8505c were transfected with control siRNA (siCtrl) or hnRNPA1-targeting siRNA (sihnRNPA1) for 48 hours, embedded in 3D collagen and treated with OTX-015 (0.5 μmol/L) for additional 48 hours. The effect on cell proliferation was determined using the WST-1 assay. Two-way ANOVA analysis was performed. ***, p<0.001 ****, p<0.0001. Error bars represent SD from three technical replicates. Results are representative of three independent experiments.

**Figure S2**: Quercetin decreases hnRNPA1 protein. **A.**Thyroid and pancreatic cancer cells were treated with Quercetin (20 μmol/L) for 24, 48, or 72 hours, and the lysates were analyzed for hnRNPA1 and GAPDH by western blot analysis. The results are representative of three biological replicates. **B.** Thyroid and pancreatic cancer cells, treated with increasing concentrations of Quercetin for 24 hours, were analyzed for hnRNPA1 by qPCR. Error bars represent SEM from three independent experiments.

**Figure S3**: Co-treatment of Quercetin and JQ1 decreases Survivin while having no effects on other apoptosis regulating proteins. CD18 and K1 cancer cells were treated with DMSO, JQ1 (1 μmol/L), Quercetin (20 μmol/L), or a combination of JQ1 (1 μmol/L) and Quercetin (20 μmol/L) for 24 hours. Cell lysates were collected and analyzed for apoptosis-related proteins using the Proteome Profiler Human Apoptosis Array ARY009, and the pixel density for Bad, Bax, Bcl-2, and Survivin were quantified by ImageJ. Error bars represent SD from two technical replicates. The results are representative of two independent experiments.

**Figure S4:** : The combination treatment of Quercetin and BET inhibitor JQ1 was well-tolerated. Mice were weighed daily, and the effect of inhibitors on mouse weight at the end of treatment was compared with weight at the start of treatment.

**Figure S5:** Representative IHC images for cleaved caspase-3 and Ki67 for CD18 tumors. CD18 xenograft tumors were stained for Ki67 and cleaved caspase-3 as described in Materials and Methods. Images were taken at 10X.

**Table S1:** Calculations for coefficients of drug interaction.

**Table S2**: Effect of hnRNPA1 knockdown on JQ1-mediated changes in proteins in the ARY009 apoptosis array.

**Table S3:** Effect of Quercetin on JQ1-mediated changes in proteins in the ARY009 apoptosis array.
