# Supplementary figures and images for: Quercetin Enhances the Anti-Tumor Effects of BET Inhibitors by Suppressing hnRNPA1
Source: Int J Mol Sci. 2019 Sep 2;20(17):4293. doi: 10.3390/ijms20174293 (PMC6747365; doi:10.3390/ijms20174293)

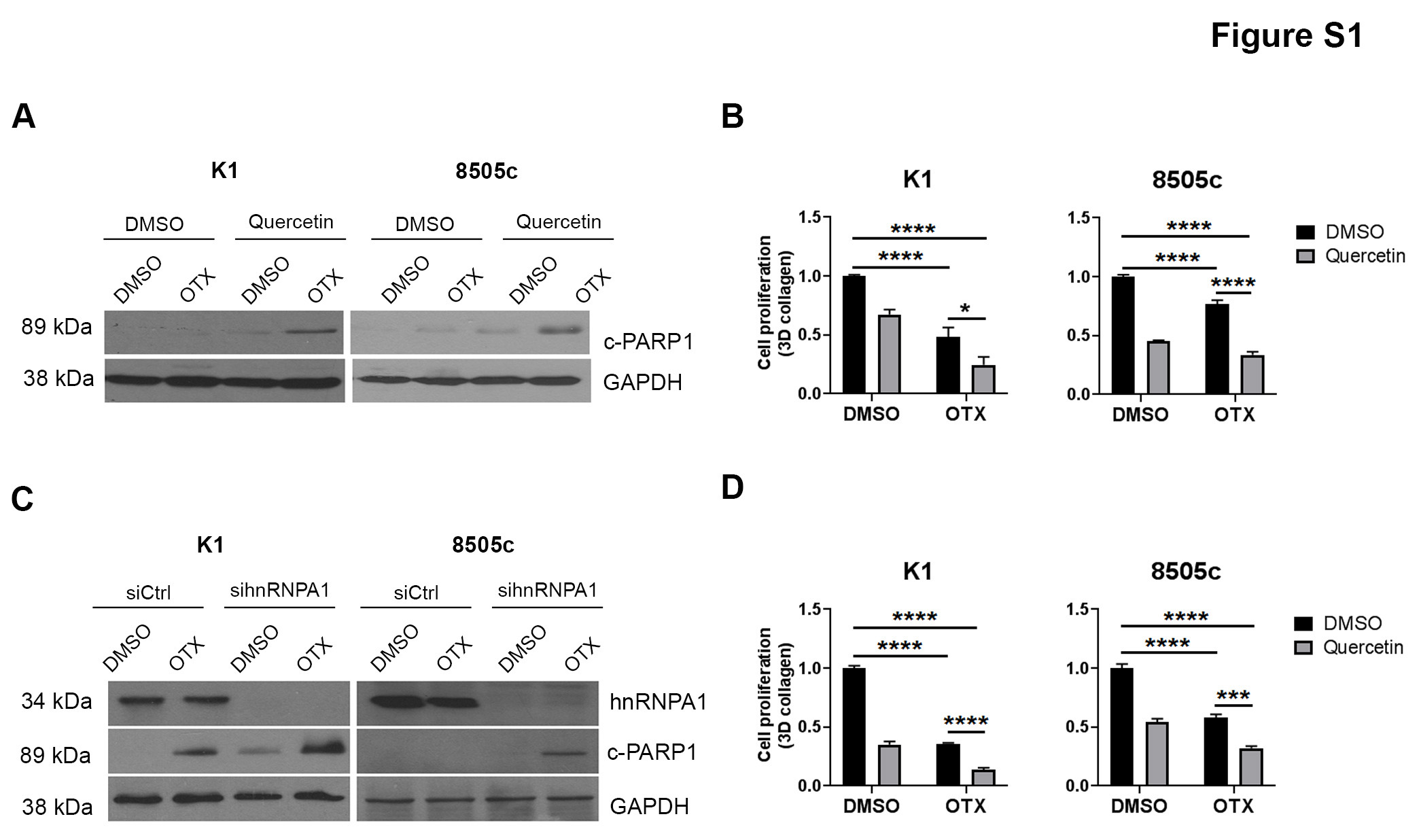

Supplement: Supplementary file 1 [file ijms-20-04293-s001.zip › ijms-558961-supplementary/Figure S1.jpg]

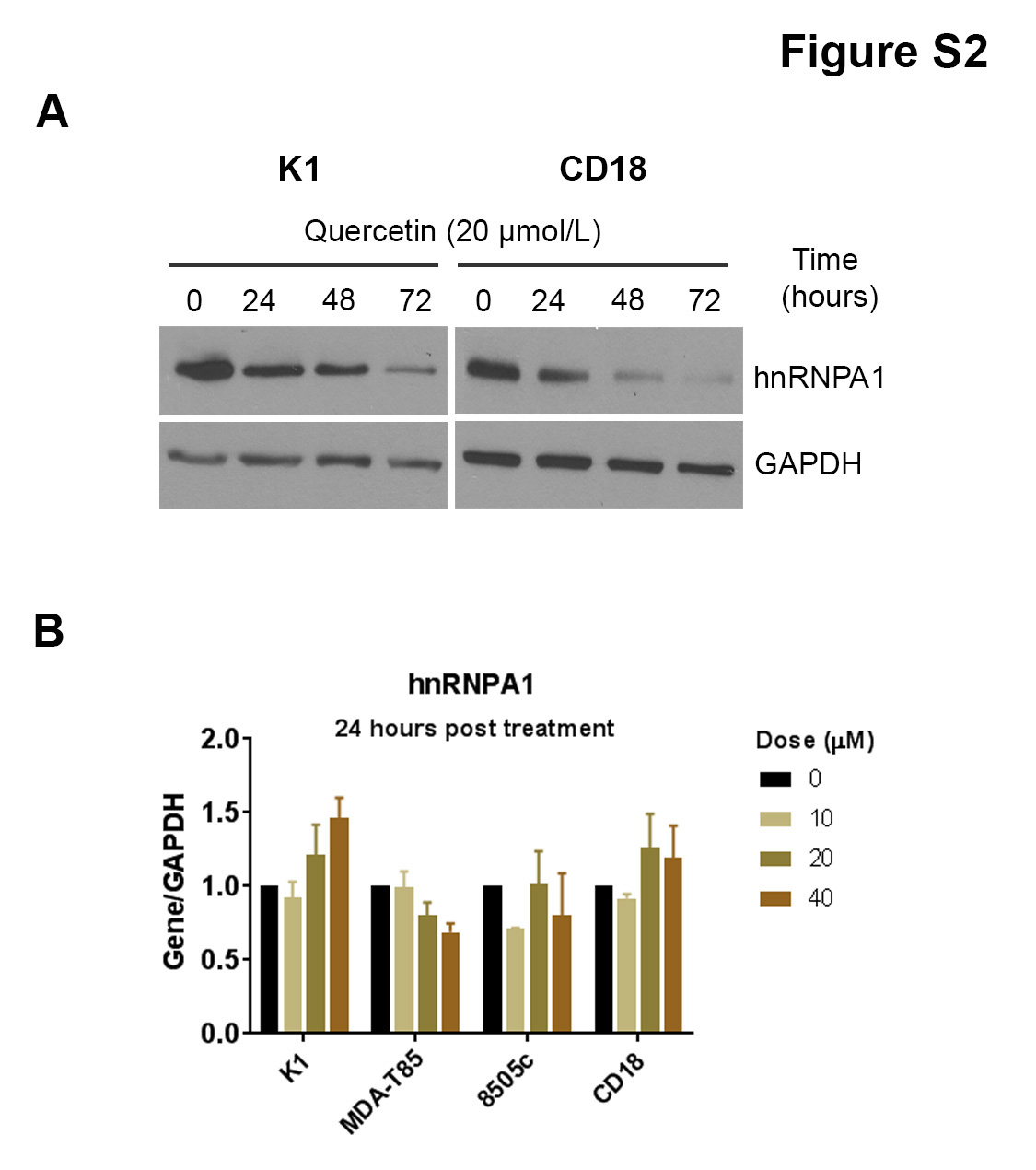

Supplement: Supplementary file 1 [file ijms-20-04293-s001.zip › ijms-558961-supplementary/Figure S2.jpg]

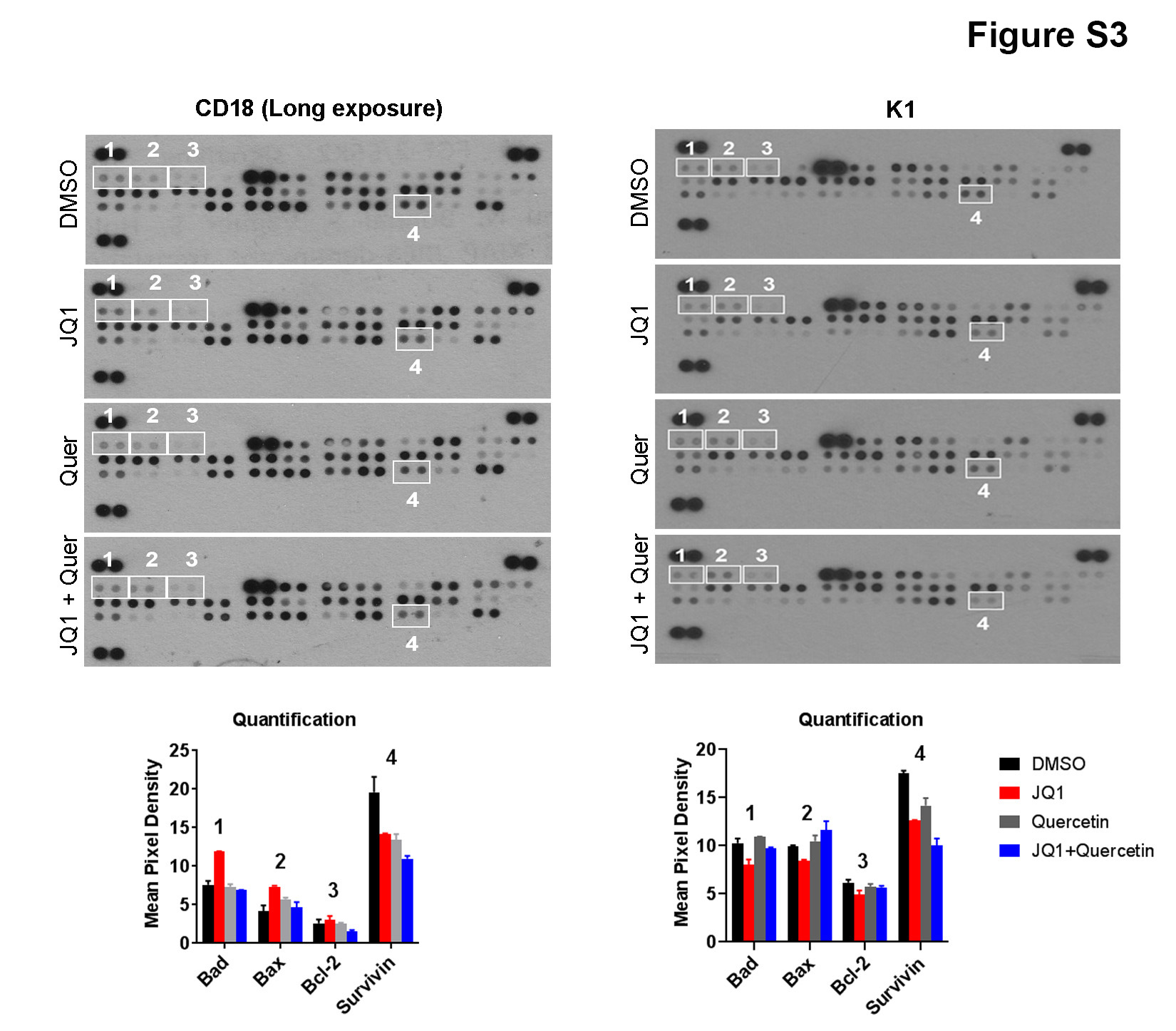

Supplement: Supplementary file 1 [file ijms-20-04293-s001.zip › ijms-558961-supplementary/Figure S3.jpg]

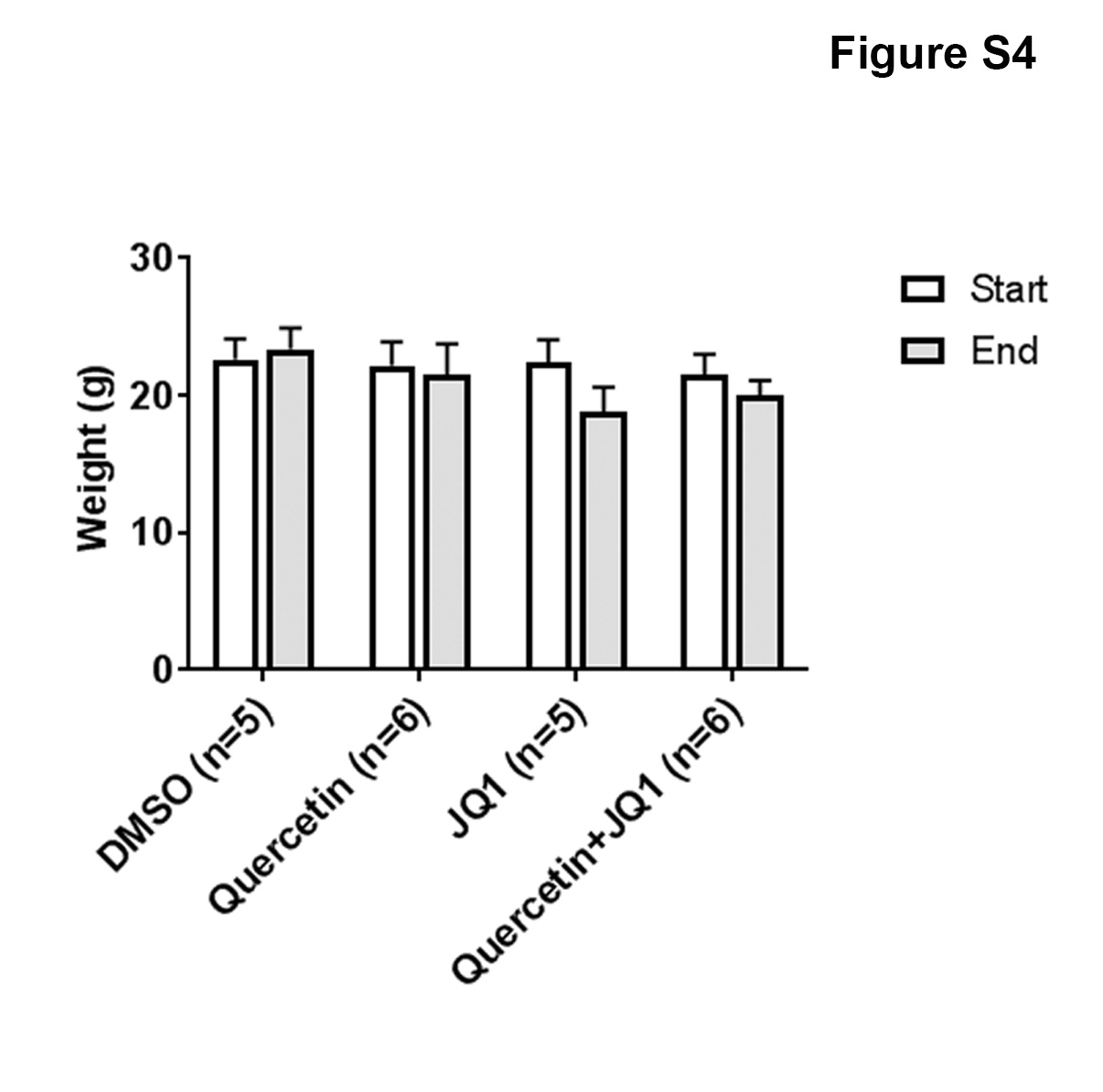

Supplement: Supplementary file 1 [file ijms-20-04293-s001.zip › ijms-558961-supplementary/Figure S4.jpg]

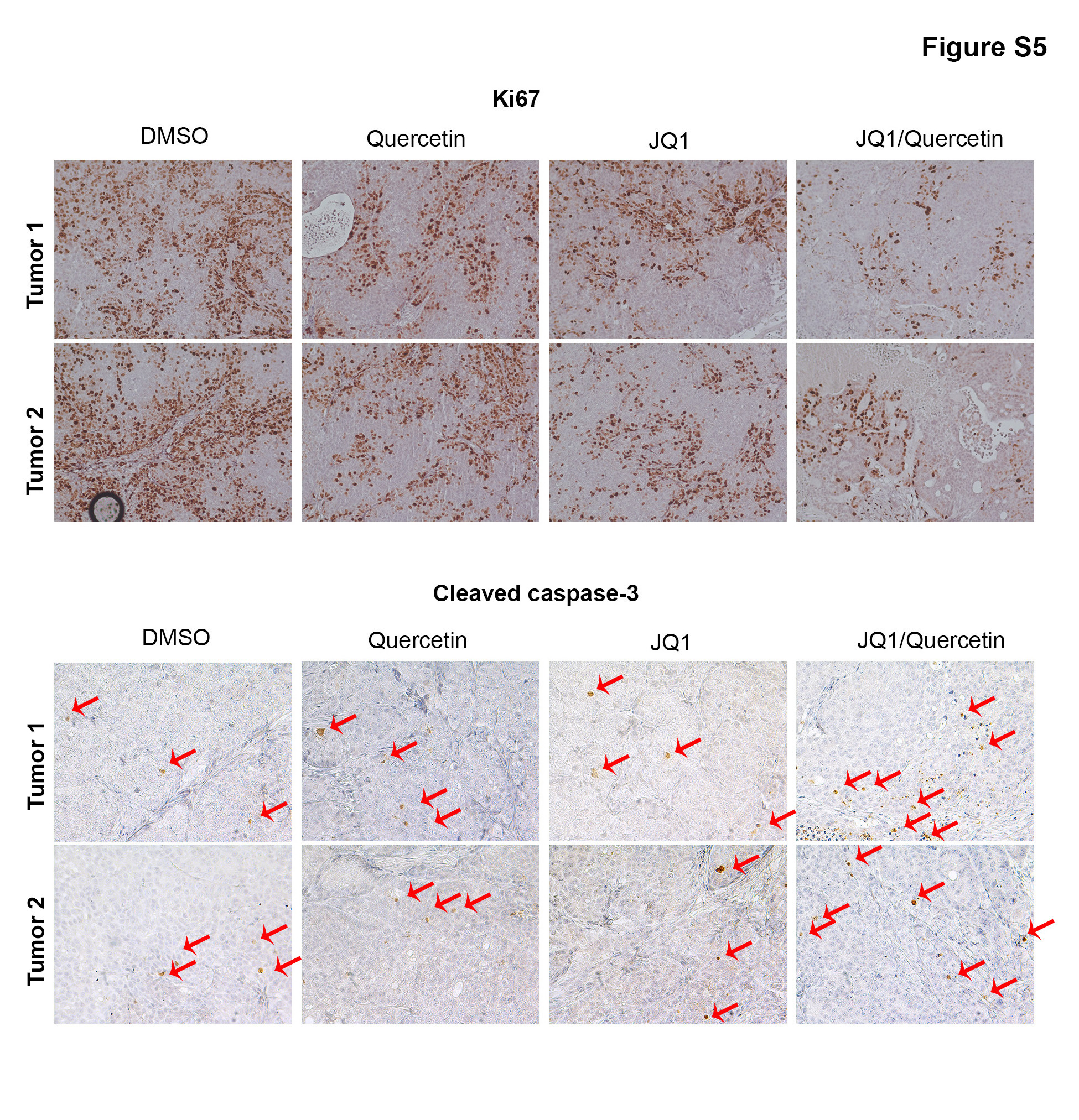

Supplement: Supplementary file 1 [file ijms-20-04293-s001.zip › ijms-558961-supplementary/Figure S5.jpg]
